# Early Transcriptome Response of *Lactococcus lactis* to Environmental Stresses Reveals Differentially Expressed Small Regulatory RNAs and tRNAs

**DOI:** 10.3389/fmicb.2017.01704

**Published:** 2017-09-14

**Authors:** Sjoerd B. van der Meulen, Anne de Jong, Jan Kok

**Affiliations:** ^1^Department of Molecular Genetics, Groningen Biomolecular Sciences and Biotechnology Institute, University of Groningen Groningen, Netherlands; ^2^Top Institute Food and Nutrition Wageningen, Netherlands

**Keywords:** RNA-Seq, sRNAs, transcriptomics, environmental stress, *L. lactis*

## Abstract

Bacteria can deploy various mechanisms to combat environmental stresses. Many genes have previously been identified in *Lactococcus lactis* that are involved in sensing the stressors and those that are involved in regulating and mounting a defense against the stressful conditions. However, the expression of small regulatory RNAs (sRNAs) during industrially relevant stress conditions has not been assessed yet in *L. lactis*, while sRNAs have been shown to be involved in many stress responses in other bacteria. We have previously reported the presence of hundreds of putative regulatory RNAs in *L. lactis*, and have used high-throughput RNA sequencing (RNA-seq) in this study to assess their expression under six different stress conditions. The uniformly designed experimental set-up enabled a highly reliable comparison between the different stress responses and revealed that many sRNAs are differentially expressed under the conditions applied. The primary stress responses of *L. lactis* NCDO712 was benchmarked to earlier work and, for the first time, the differential expression was assessed of transfer RNAs (tRNAs) and the genes from the six recently sequenced plasmids of NCDO712. Although, we only applied stresses for 5 min, the majority of the well-known specific stress-induced genes are already differentially expressed. We find that most tRNAs decrease after all stresses applied, except for a small number, which are increased upon cold stress. Starvation was shown to induce the highest differential response, both in terms of number and expression level of genes. Our data pinpoints many novel stress-related uncharacterized genes and sRNAs, which calls for further assessment of their molecular and cellular function. These insights furthermore could impact the way parameters are designed for bacterial culture production and milk fermentation, as we find that very short stress conditions already greatly alter gene expression.

## Introduction

Bacteria display many general as well as specific molecular responses to environmental changes. Sudden alterations in the environment can be of physical or chemical nature and can threaten the lifespan of a microbial cell, especially if the stress condition is too intense in time or intensity. The metabolic activity of bacteria used in industrial fermentations is altered upon stress in their for example acidification rates and flavor formation (Xie et al., 2004; Taïbi et al., 2011). Gaining insights in the effects of stress could improve the predictability, quality, and safety of fermentations.

The lactic acid bacterium (LAB) *Lactococcus lactis* is of eminent importance in the dairy industry, where it is used worldwide for the production of a large variety of cheeses and of buttermilk. The main function during milk fermentation of LAB such as *L. lactis* is to convert lactose into lactic acid. The consequent lowering of the pH leads to an increased shelf life of the fermented products as it prevents outgrowth of spoilage or pathogenic organisms. In addition, *L. lactis* provides texture, flavors, and aromas to the end products (Marilley and Casey, 2004; Smit et al., 2005). During their preparation as a starter culture, as well as in the actual fermentation process, fluctuations in temperature, osmolarity, pH, and nutrient availability cause significant stress to the *L. lactis* cells. They have evolved different response systems to sense and respond to potential lethal conditions and to defend themselves accordingly, in order to survive. Many of these mechanisms and the regulatory systems involved have been identified in *L. lactis*, on the basis of homology to proteins with known functions in other organisms and/or by experimental validation (Sanders et al., 1999; Smith et al., 2010). The potential role in stress of small non-coding regulatory RNAs (sRNAs) has not yet been assessed in *L. lactis*, while sRNAs have been shown to play an important function in a variety of stress conditions in other bacteria (Gottesman et al., 2006; Romby and Charpentier, 2010; Hoe et al., 2013).

Bacterial regulatory RNAs are generally non-coding transcripts that modulate gene expression post-transcriptionally (Waters and Storz, 2009). They are usually classified on whether or not genes are encoded on the strand opposite to the strand from which they derive. Non-coding RNAs that are located within intergenic regions (IGRs) are referred to as small RNAs (sRNAs) and roughly contain between 50 and 350 nucleotides. They are very heterogeneous in size and structure, and act in *trans* to target mRNAs (Gottesman and Storz, 2011; Storz et al., 2011). Transcripts that overlap in an antisense fashion with mRNAs from the opposite strand are called antisense RNAs (asRNAs) (Thomason and Storz, 2010; Georg and Hess, 2011). sRNAs and asRNAs with proven functions in regulating other RNAs and/or proteins can be considered regulatory RNAs. Base-pairing between a regulatory RNA and its target mRNA(s) can affect mRNA stability as well as translation, the latter by influencing the accessibility of the ribosomal binding site (RBS) on the target transcript (Morita and Aiba, 2011; Prevost et al., 2011; Bandyra et al., 2012; Papenfort and Vanderpool, 2015). Since the discovery of the first regulatory RNAs, starting with antisense RNAs involved in plasmid copy number control (Stougaard et al., 1981; Tomizawa et al., 1981) and the first genomically encoded sRNA MicF (Mizuno et al., 1984), many others have been described especially since recent advances have been in high-throughput RNA sequencing (RNA-seq) as well as in high-density tilling arrays (Nicolas et al., 2012). Various mechanisms of action have been elucidated since but determining the functions and mechanisms of action of novel sRNAs is still the major challenge. This may be illustrated by the abundant non-coding RNA 6S, which was discovered as early as 1967 (Hindley, 1967) but of which the function in modifying RNA polymerase activity was uncovered only in 2000 (Wassarman and Storz, 2000).

Several examples exist of sRNAs that perform a crucial role in the adaptation and survival of bacteria during stressful conditions. For instance, the sRNA RybB from *E. coli* and *Salmonella* is activated by the extracytoplasmic stress sigma factor, σ^E^. Accumulation of misfolded outer membrane proteins (OMPs), for example in the stationary phase, can cause cell envelope stress. RybB downregulates many different *omp* mRNAs in order to prevent the synthesis of OMPs and to restore envelope homeostasis (Johansen et al., 2006; Papenfort et al., 2006; Papenfort and Vogel, 2009). Another striking example of how effective and diverse the scope of one sRNA can be is the bifunctional sRNA SgrS from *E. coli* and *Salmonella*. The transcription factor SgrR is triggered upon glucose-phosphate stress and activates SgrS transcription. The stress is immediately relieved by detoxification of the phosphosugar. This is mediated by SgrS base-pairing with *yigL*, blocking *yigL* degradation by RNase E and resulting in increased YigL expression (Papenfort et al., 2013). To decrease phosphosugar accumulation in the cell by PtsG and ManXYZ, SgrS base-pairs with and blocks translation of the mRNAs of *manXYZ* and *ptsG*, which are eventually degraded (Bobrovskyy and Vanderpool, 2014). The 5′ end of SgrS contains an ORF, *sgrT*. This SgrT peptide interacts with PtsG in such a way that it blocks the uptake of glucose (Wadler and Vanderpool, 2007).

Recently, we have re-annotated the genome of *L. lactis* MG1363 by extensive mining of differential RNA sequencing (dRNA-seq) data of samples taken at various points during growth as a batch culture (van der Meulen et al., 2016). This has led to the addition of 186 small non-coding RNAs and 60 antisense RNAs to the *L. lactis* genome annotation. Here we have treated the parent strain of *L. lactis* MG1363, *L. lactis* NCDO712, which carries a number of plasmids with industrial relevance (Tarazanova et al., 2016), to a number of industrial stresses. Specifically, the strain was exposed for a relatively short period of time of 5 min to cold, heat, acid, osmotic, oxidative or starvation stress to explore the organism's early transcriptome responses by strand-specific RNA-seq. The expression of recently annotated sRNAs and asRNAs was assessed and a significant number of them were observed to be differentially expressed under the various stress conditions. Moreover, extensive data mining allowed pinpointing many genes that are involved in the investigated, industry-related stress conditions.

## Materials and methods

### Bacterial strains and media

*Lactococcus lactis* subsp. *cremoris* NCDO712 is an industrial strain containing six plasmids of which one, pLP712, carries lactose and casein utilization genes (Tarazanova et al., 2016). *L. lactis* MG1363 is its plasmid-free derivative (Gasson, 1983). *L. lactis* NCDO712 was grown as a standing culture at 30°C in M17 broth (Difco, Becton Dickinson, Le Pont de Claix, France) containing 0.5% (w/v) glucose (GM17), and on GM17 agar plates.

### Stress treatments

All stress conditions were applied in two biological replicates by starting with two single colonies of *L. lactis* NCDO712 grown on GM17 agar plates. These were each used to inoculate 10 ml of fresh GM17 media and grown overnight. Each overnight culture was diluted 100-fold in a bottle with 500 ml GM17 and grown until an optical density at 600 nm (OD_600_) of 0.9 was reached. The content of each bottle was divided over seven 50-ml Greiner tubes and centrifuged at 4,000 g for 1.5 min. Subsequently, the cultures from each bottle were subjected to all conditions tested. The cell pellets were re-suspended in fresh GM17 containing 0.25% glucose (G^*^M17) and the following properties: control (G^*^M17 at 30°C), cold (G^*^M17 at 10°C), heat (G^*^M17 at 42°C), acid (G^*^M17 set at pH 4.5 with lactic acid), osmotic stress (G^*^M17 containing 2.5% NaCl), and oxidative stress (G^*^M17, shaking at 250 rpm). The cold stress was applied in an incubator while the heat stress was performed in a water bath to maintain the cold and heat conditions stable. For starvation stress, the cell pellets were re-suspended in filter-sterilized phosphate-buffered saline (PBS). The stress conditions were applied for 5 min, after which the cells were harvested by centrifugation at 10,000 rpm for 1 min, snap frozen in liquid nitrogen and stored at −80°C prior to RNA isolation.

### RNA isolation

RNA was isolated as described previously (van der Meulen et al., 2016). All procedures were executed at 4°C unless otherwise stated and all solutions were DEPC-treated and subsequently autoclaved. Frozen cell pellets were re-suspended in 400 μl TE-buffer (10 mM Tris-HCl, 1 mM EDTA, pH 7.4) and added to 50 μl 10% sodium dodecyl sulfate (SDS), 500 μl phenol/chloroform (1:1 v/v), and 0.5 g glass beads (75–150 μm, Thermo Fischer Scientific, Rockford, IL, United States). The cells were disrupted by shaking 2 times for 45 s in a Biospec Mini-BeadBeater (Biospec Products, Bartlesville, OK, United States) with cooling on ice for 1 min between the shaking steps. Subsequently, the cell suspension was centrifuged at 14,000 rpm for 10 min. The upper phase containing the nucleic acids was treated with 500 μl chloroform and centrifuged as above. Nucleic acids in the water phase were precipitated by sodium acetate and ethanol. The nucleic acid pellet was re-suspended in 100 μl buffer consisting of 82 μl MQ, 10 μl 10x DNase I buffer, 5 μl RNase-free DNase I (Roche Diagnostics GmbH, Mannheim, Germany), and 3 μl RiboLock RNase inhibitor (Fermentas/Thermo Scientific, Vilnius, Lithuania), and treated for 30 min at 37°C. The RNA was then purified using standard phenol/chloroform extraction and sodium acetate/ethanol precipitation. RNA pellets were re-suspended in 50 μl elution buffer from the High Pure RNA Isolation Kit (Roche Diagnostics, Almere, the Netherlands) and stored at −80°C.

### RNA treatment, library preparation, and RNA deep sequencing

RNA concentration was measured with a Nanodrop ND-1000 (Thermo Fischer Scientific). As a measure of RNA quality, the integrity of the 16S/23S rRNA and the presence of any DNA contamination were assessed by using an Agilent 2100 Bioanalyser (Agilent Technologies, Waldbronn, Germany). cDNA library was prepared by employing a ScriptSeq^TM^ Complete Kit for Bacteria (Epicentre, Madison, WI, United States) including Ribo-Zero™ for rRNA removal. The cDNA libraries were sequenced at Otogenetics Corporation (Norcross, GA, United States) on an Illumina HiSeq2000.

### Data analysis

Raw sequence reads were analyzed for quality and trimmed with a PHRED score >28. Read alignment was performed on the genomic DNA of *L. lactic* NCDO712 (nucleotide sequences of the chromosome and all six plasmids of NCDO712; Tarazanova et al., 2016) using Bowtie 2 (Langmead and Salzberg, 2012). RKPM values were used as an input for the T-REx analysis pipeline (de Jong et al., 2015) together with a text file describing the factors, contrasts, and classes. In the class file, genes from the NCDO712 plasmids were colored green while sRNAs were colored red (see Supplementary Information S1). T-REx was used to perform all statistical analyses (de Jong et al., 2015).

### Data access

The RNA-seq data is publically available in GEO under accession number GSE98499.

## Results

### RNA-seq reveals that starvation has a large impact on the transcriptome of *L. lactis*

**Figure 1:**
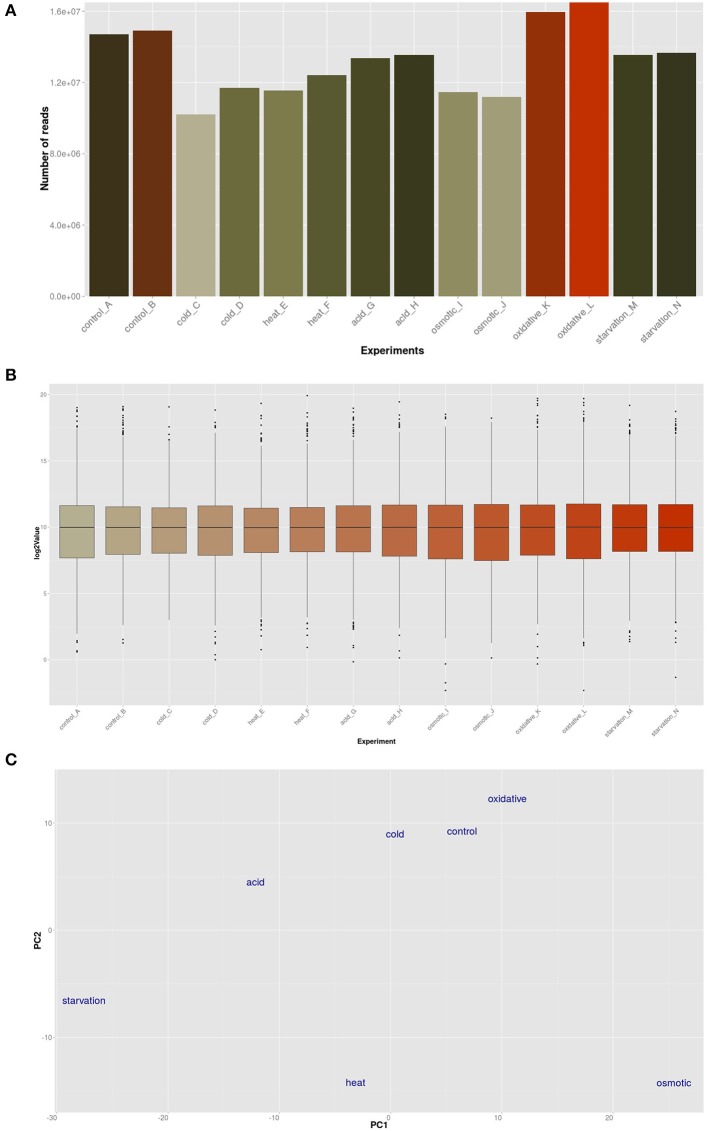
Global analysis by T-Rex (de Jong et al., 2015) of the RNA-seq data. **(A)** Total number of RNA-seq reads per sample; **(B)** Box-plot expression values of all experiments; **(C)** Principle Component Analysis (PCA) plot of the conditions employed in this study.

**Figure 2:**
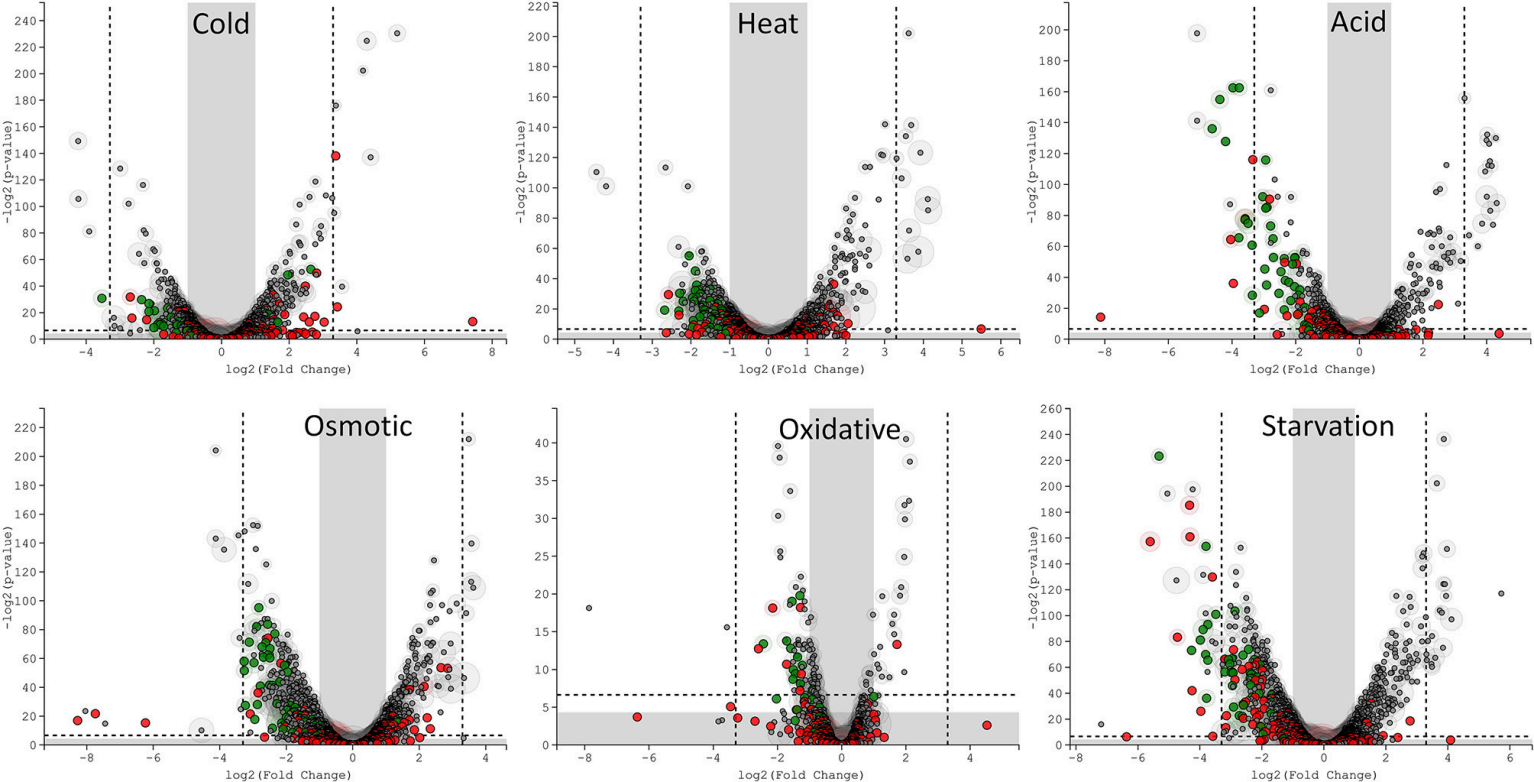
T-REx-generated Volcano plots of the six different stress conditions displaying significance vs. gene expression on the y and x axes respectively. Outside the gray areas: genes with fold change ≥ 2 and a *p* ≤ 0.05, outside the dashed lines: genes with fold change ≥ 5 and a *p* ≤ 0.01. Green circles: tRNA genes, red circles: sRNA genes, gray circles: all other genes.

**Figure 3:**
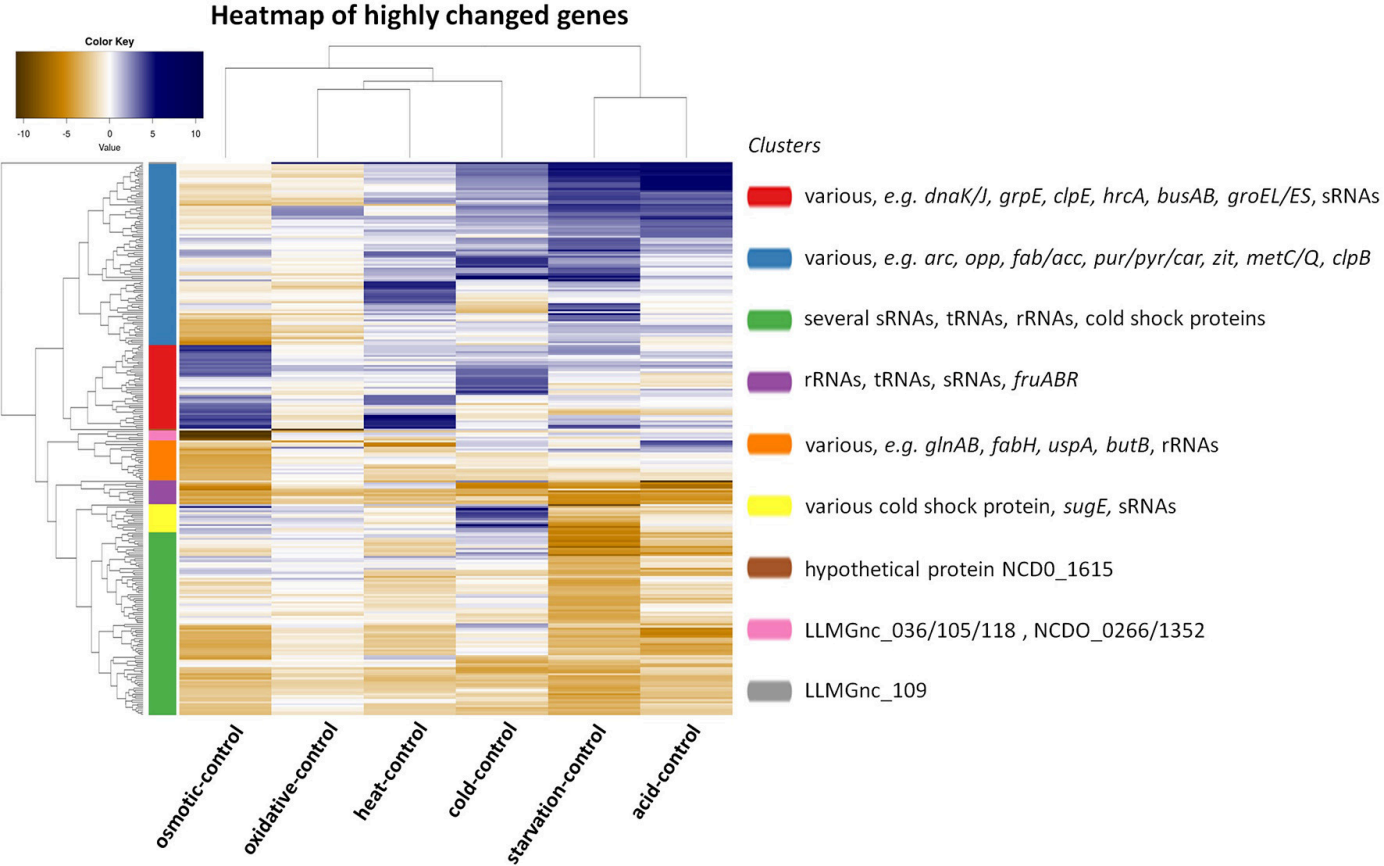
Heat map showing the correlation of a comparison between the indicated stress condition vs. its control and clusters of genes of which the expression has changed with a high fold (fold change ≥ 5 and *p* ≤ 0.01). Nine clusters were calculated by T-REx (de Jong et al., 2015) and dominant and stress-related genes are listed in clusters.

The transcriptomic response of *L. lactis* subsp. *cremoris* NCDO712 (hereafter named *L. lactis* NCDO712) after 5 min of cold, heat, acid, osmotic, oxidative, or starvation stress was determined by high-throughput RNA sequencing. This resulted in a total of 246M of reads of which 209M reads (85%) were successfully mapped on the genome and plasmids of *L. lactis* NCDO712. The libraries varied between 11M and 19M reads per individual sample (Figure 1A). The data was normalized using the T-REx software (de Jong et al., 2015) and plotted in a box plot of normalized signals for all samples (Figure 1B). A Principle Component Analysis (PCA) shows that the stress conditions are statistically well-distributed from each other (Figure 1C). The transcriptome of cells exposed to osmotic stress or to starvation were most different from that of the control. The absolute numbers of differentially expressed genes underpin these observations; the largest transcriptome changes were observed after starvation (756 genes involved), while oxidative stress had the least impact (91 affected genes). Table 1 gives an overview of the counts of the differentially expressed genes and Figure 2 shows the distribution of affected genes for each stress condition. To gain insight in the distribution of the stress-responsive genes, those of which the expression changed highly (fold change ≥ 5 and *p* ≤ 0.01) under all conditions were visualized in a heatmap. T-REx was used to pinpoint nine clusters that vary strongly in size and in the functions of the constituting genes. One cluster is rich in stress-related genes, while another one contains predominantly sRNAs. See Figure 3 for the heatmap and the complete list of cluster descriptions. Genes that were affected by a fold change ≥ 10-fold and *p* ≤ 0.01) are listed in Table 2 and are discussed in more detail below.

**Table 1:** Absolute numbers of differentially expressed genes after 5 min of exposure to the indicated stress.

**Stress condition**	**Upregulated genes**		**Downregulated genes**		**Total affected genes**	
	High fold^*^	**Top hits^#^**	**High fold**	**Top hits**	**High fold**	**Top hits**
Cold	36	240	15	172	51	412
Heat	27	249	8	244	35	493
Acid	37	126	40	190	77	316
Osmotic	30	268	68	433	98	701
Oxidative	0	23	4	68	4	91
Starvation	55	325	95	431	150	756

* *Genes with a fold change ≥ 5 and a p ≤ 0.01*.

# *Genes with a fold change of ≥ 2 and a p ≤ 0.05*.

**Table 2:** Genes differentially expressed as a result of various stress conditions.

**Locus tags**		**Gene: description**	**Upregulation or downregulation**					
**NCDO712**	**MG1363**		**Cold**	**Heat**	**Acid**	**Osmotic**	**Oxidative**	**Starvation**
**AMINO ACID TRANSPORT AND METABOLISM**								
NCDO_0301	llmg_0335	*plpA/metQ*: Methionine ABC transporter			6.7			3.0
NCDO_1706	llmg_1776	*metC*: Cystathionine gamma-lyase	3.1	2.7	13.2		2.8	5.6
NCDO_1707	llmg_1775	*cysK*: Cysteine synthase	4.1	2.5	10.9		2.2	7.2
NCDO_2243/7	llmg_2309/13	*arcABD1C1C2*: arginine deiminase pathway		10.9 ± 2.1			−2.8 ± 0.7	2.2 ± 0.6
NCDO_2384	llmg_2477	*lysP*: Lysine-specific permease	−8.0		−2.1			−4.9
**TRANSPORTERS, ABC/PTC/PORIN**								
NCDO_0184	llmg_0454	*bglP: PTS system. trehalose-specific IIB component*				−17.4	−2.4	3.2
NCDO_0191	llmg_0446	*msmK: Multiple sugar ABC transporter. ATP-binding protein*		3.0		−4.4	−3.8	15.4
NCDO_0199	llmg_0438	*ptcA: PTS system. cellobiose-specific IIA component*	−2.4			−14.6		
NCDO_0381	llmg_1097	*glpF2:* glycerol uptake facilitator				−10.3		2.1
NCDO_0584	llmg_1210	*emrB*: Drug resistance transporter EmrB/QacA subfamily		6.1				
NCDO_0687	llmg_0697	*oppD*: Oligopeptide transport ATP-binding protein	4.6		3.0	2.6		14.6
NCDO_0938	llmg_0910	*amtB*: Ammonium transporter				−6.7		2.2
NCDO_1110	llmg_1195	*ribU*: Substrate-specific component of riboflavin transporter	11.8		2.4			4.4
NCDO_2331/3	llmg_2398/400	*zit*operon*:* Zinc ABC transporter	13.4 ± 5.4					5.0 ± 3.5
**CARBON METABOLISM**								
NCDO_0183	llmg_0455	*trePP*: Trehalose 6-phosphate phosphorylase	6.8	2.4		−2.4	−2.5	9.8
NCDO_1543/5	llmg_1568/70	*fruAKR*: fructose operon	−17.6 ± 2.2		−28.4 ± 10	−11.1 ± 6.0		−11.9 ± 2.7
**FATTY ACID BIOSYNTHESIS**								
*accABCD*		Acetyl-CoA carboxylases	3.9 ± 1.0		6.5 ± 1.7		3.4 ± 0.4	8.0 ± 2.8
*fabIZ1THDGFZ2*		Fatty acid biosynthesis	2.0 ± 0.9	−2.1 ± 0.9	3.7 ± 1.6	−3.2 ± 1.5	3.7 ± 0.7	1.9 ± 3.0
**NUCLEOTIDE TRANSPORT AND METABOLISM**								
*purFLQSCHD*		*de novo biosynthesis of purines*	2.7 ± 0.5	2.0 ± 0.4	7.9 ± 5.2			6.5 ± 2.4
*pyrRPBKDBFEC-carAB*		*de novo biosynthesis of pyrimidines*	4.3 ± 0.9		16.9 ± 1.7			12.5 ± 3.5
**STRESS RESPONSE**								
NCDO_0206	llmg_0430	*cstA*: Carbon starvation protein A				−2.3		2.5
NCDO_0207	llmg_0429	*sodA*: Manganese superoxide dismutase					−1.9	−2.1
NCDO_0225	llmg_0411	*groEL*: Heat shock protein 60 family chaperone		17.4		7.3	−2.1	
NCDO_0226	llmg_0410	*groES*: Heat shock protein 60 family co-chaperone	−2.0	17.3		7.5	−2.4	
NCDO_0463	llmg_0180	*cspE*: cold shock protein E		−2.2		−3.1		−9.1
NCDO_0514	llmg_0132	*sugE*: Quaternary ammonium compound-resistance protein	18.2					−2.3
NCDO_0544	llmg_0528	*clpE: ATP-dependent Clp protease. ATP-binding subunit*		12.4		6.0		2.1
NCDO_0641	llmg_0638	*clpP: ATP-dependent Clp protease. proteolytic subunit*	2.0	3.7		4.4		
NCDO_0951	llmg_0986	*clpB: Chaparonin. ClpB protein*	3.0	6.0		3.0		2.8
NCDO_1029	llmg_1049	*busAB*: ABC-type glycine betaine transport system			−2.2	6.7		−2.6
NCDO_1030	llmg_1048	*busAA: Glycine betaine ABC transport system. ATP-binding*			−2.7	7.7		−4.7
NCDO_1062	llmg_1104	*lmrB*: Multidrug resistance protein B	2.7	−4.1	−2.3	−6.3		−2.7
NCDO_1185	llmg_1256	*cspD*: cold shock protein D	4.2					−5.2
NCDO_1186	llmg_1255	*cspC*: cold shock protein C	7.4			2.5		−7.7
NCDO_1206	llmg_1256	*cspD*: cold shock protein D		−2.3	−3.3			−27.0
NCDO_1537	llmg_1576	*hrcA*: Heat-inducible transcription repressor		10.8	3.1	11.8		8.8
NCDO_1538	llmg_1575	*grpE*: Heat shock protein		15.2		12.4		6.7
NCDO_1539	llmg_1574	*dnaK*: chaperone protein		14.6		10.2		2.3
NCDO_1794	llmg_1847	*cspA*: cold shock protein A	21.2			3.1		−14.1
NCDO_1795	llmg_1846	*cspB:* cold shock protein B	7.7					−33.1
NCDO_2408	llmg_2502	*dnaJ*: Chaperone protein	−3.5	5.3		4.9		
**TRANSCRIPTION FACTORS**								
NCDO_0643	llmg_0640	*spxA*: transcriptional regulator Spx/MgsR		−3.2	−3.6			−18.8
NCDO_0937	llmg_0911	*glnB*: Nitrogen regulatory protein P-II		−2.8	2.1	−10.8		3.5
NCDO_1088	llmg_1130	*spxA*: Transcriptional regulator Spx/MgsR		2.4		3.0		52.9
NCDO_1129	llmg_1177	*gadR*: positive regulator	−6.7					−4.0
NCDO_1636	llmg_1703	*spxA*: transcriptional regulator Spx	−4.7	3.0				12.5
NCDO_1781	llmg_1860	*rmaB: Transcriptional regulator. MarR family*	−2.0	7.5				
NCDO_2330	llmg_2401	*zitR*: Transcriptional repressor AdcR for Zn(2+)	36.6		2.6			15.7
**UNKNOWN FUNCTION OR POORLY CHARACTERIZED**								
NCDO_0342	llmg_0294	Hypothetical protein	−3.1		−4.0	−3.3	−2.0	−12.2
NCDO_1187	llmg_1254	Hypothetical protein directly upstream of cspC	8.5					−3.3
NCDO_1519	llmg_1594	*yjgB*: P54 ENTFC NLPP60 family protein	5.0	−3.2	-3.4	11.2		−5.5
NCDO_1793	llmg_1848	Hypothetical protein directly downstream of cspA	9.7		-2.8			−4.4
NCDO_2075/7	llmg_2163/4	*yth*operon: hypothetical proteins				10.4 ± 1.6		3.3 ± 0.3
NCDO_2419	llmg_2513	*ywoG*: Major facilitator family transporter	3.0	−4.5	9.1	−6.6	−3.4	11.2
NCDO_2420	llmg_2514	SH1215: Universal stress protein family		−18.3	6.8	−3.2		
NCDO_2421	llmg_2515	Hypothetical protein		−21.7	4.2	−3.2		

*Genes are shown in this table when they are differentially expressed ≥10-fold, with a p ≤ 0.05 and logCPM ≤ 1 in at least one of the stress conditions, or previously reported in the literature during the relevant stress condition. sRNA and tRNA genes are excluded from this table as they are reported separately*.

### sRNAs are highly affected after 5 min of stress induction

**Figure 4:**
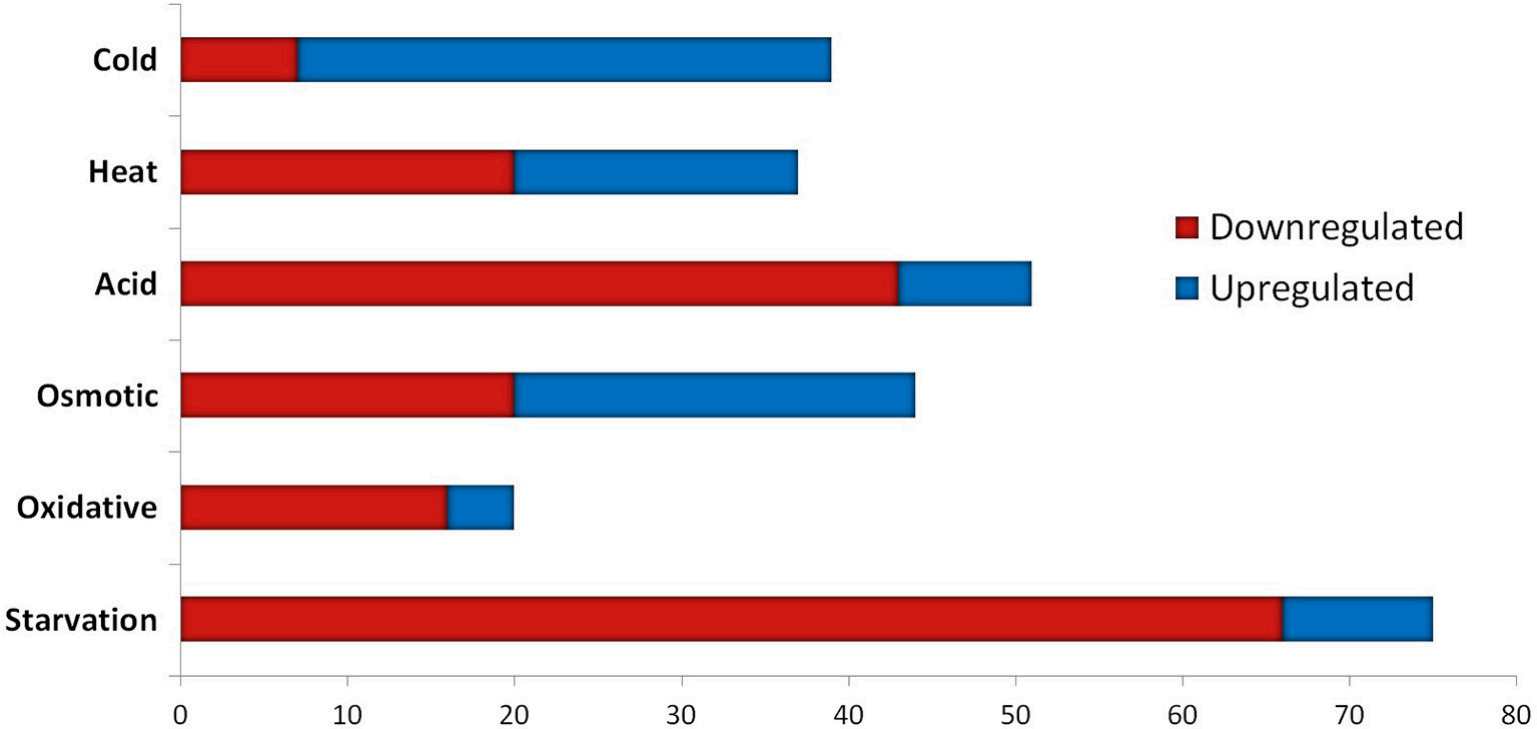
Overview of significantly differentially expressed sRNA genes under various stress conditions (cut-off fold change ≥ 2, *p* ≤ 0.05 and logCPM > 1).

We assessed the expression of the 186 sRNAs that have recently been identified in the genome of *L. lactis* MG1363 (van der Meulen et al., 2016). The majority of the sRNAs that were significantly changed after applying acid, oxidative, or starvation stress showed a decrease in expression. In contrast, after cold stress more sRNAs were upregulated instead of downregulated (see Figure 4). Of the 186 sRNAs, the expression of 110 was significantly changed after applying at least one stressor, while 42 sRNAs were differentially expressed under at least three stress conditions (Table 3). This list of sRNAs was restricted to those with a logCPM value >1. The expression of some of these 110 sRNA genes was highly affected under only one specific stress condition. For example, LLMGnc_152 (10.7-fold) and LLMGnc_153 (6.0-fold) were increased specifically after cold stress, while LLMGnc_176 (6.3-fold) showed a higher expression after salt stress. The sRNA LLMGnc_092 (−9.2-fold) was only decreased after starvation, while LLMGnc_025/064/065/073 were downregulated in all conditions.

**Table 3:** Differential expression of sRNA genes under industrially relevant stress conditions.

**Small RNA**	**Fold change per condition**						**Small RNA**	**Fold change per condition**					
	**Cold**	**Heat**	**Acid**	**Osmotic**	**Oxidative**	**Starvation**		**Cold**	**Heat**	**Acid**	**Osmotic**	**Oxidative**	**Starvation**
LLMGnc_001	3.3			2.2			LLMGnc_086	2.5				−2.1	
LLMGnc_002			−3.0		−2.2	−5.8	LLMGnc_087		−3.5	−3.4			−15.7
LLMGnc_005		3.2				2.7	LLMGnc_088			−2.3			−5.3
LLMGnc_006	4.6		2.3		−4.6		LLMGnc_091			−3.8			−2.3
LLMGnc_009		−2.0				−3.8	LLMGnc_092						−9.2
LLMGnc_010						−4.0	LLMGnc_093			−2.2		−2.0	−6.1
LLMGnc_013	3.1	2.3	2.5	2.4			LLMGnc_095						−2.3
LLMGnc_015		2.5	−2.4	2.1			LLMGnc_098						−2.1
LLMGnc_016				2.3			LLMGnc_099	3.7					−4.7
LLMGnc_017				−3.1		−2.1	LLMGnc_100	3.0				−2.1	
LLMGnc_018	2.6						LLMGnc_102	2.3	2.0				−2.9
LLMGnc_019	5.4	2.1	2.0	2.1	−9.4	2.9	LLMGnc_103						−4.2
LLMGnc_022			−5.1			−7.5	LLMGnc_106			−2.1			−2.7
LLMGnc_023	6.8	4.2				6.9	LLMGnc_109			−2.1			−2.8
LLMGnc_025	−4.6	−5.0	−4.8	−3.1	−2.7	−6.0	LLMGnc_110		−2.6		−2.7		−2.6
LLMGnc_026	2.7			4.7			LLMGnc_111						−2.1
LLMGnc_027				2.1			LLMGnc_112	−2.1		−2.3		−2.2	
LLMGnc_029		2.6					LLMGnc_113						−2.8
LLMGnc_031	4.5	−2.3				−12.0	LLMGnc_114		−2.0	−3.0	−2.4		−3.0
LLMGnc_032			−2.5			−4.6	LLMGnc_115				−2.7		−2.5
LLMGnc_034	4.9	3.8	2.5		2.5	5.2	LLMGnc_118		−4.1		−75.3		
LLMGnc_035	2.2	2.2	−2.3	2.8	3.3	−2.6	LLMGnc_119						−3.7
LLMGnc_036				−214			LLMGnc_121			5.6			
LLMGnc_038	2.1	3.2		3.6			LLMGnc_127			−2.2			
LLMGnc_039		−2.3	−2.5			−4.1	LLMGnc_128	6.4	3.4	−6.0	3.3		
LLMGnc_042			−3.0				LLMGnc_129		−2.1				
LLMGnc_046			−3.6			−7.7	LLMGnc_130	2.1			−2.2		
LLMGnc_047	5.6		−2.6	7.2			LLMGnc_131			−10.2			−4.7
LLMGnc_048						−2.6	LLMGnc_132		−3.3				−9.0
LLMGnc_049	2.7		2.1			3.8	LLMGnc_137				2.0		
LLMGnc_055						−2.3	LLMGnc_138				−2.5	−3.3	−4.5
LLMGnc_056		2.7			2.1		LLMGnc_141	8.2	2.2		2.2		
LLMGnc_057			−7.1			−20.1	LLMGnc_142			−2.1	−2.3		−3.1
LLMGnc_058			−3.9			−12.1	LLMGnc_143			−2.0	−6.3	−2.1	−2.3
LLMGnc_059		−2.4					LLMGnc_145			−2.2			−7.3
LLMGnc_060						2.6	LLMGnc_147			−2.9	−3.4		−2.3
LLMGnc_061			−2.4	−2.3	−2.5	−2.4	LLMGnc_148	−2.5	−2.1		−5.9		−3.2
LLMGnc_062		−3.1	−15.6	−4.1		−4.9	LLMGnc_149			−2.8			
LLMGnc_064	−6.3	−3.8	−7.9	−8.5	−6.0	−8.8	LLMGnc_150						−4.4
LLMGnc_065	−6.5	−6.0	−16.5	−7.2	−4.4	−26.4	LLMGnc_152	10.7					
LLMGnc_066		−2.0	−2.2			−20.0	LLMGnc_153	6.0					
LLMGnc_068						−2.6	LLMGnc_155			−3.0	3.3		−3.1
LLMGnc_069	2.9					−2.0	LLMGnc_157			−2.1			−4.3
LLMGnc_070			2.4			2.0	LLMGnc_161	5.4		−2.1			
LLMGnc_072				4.4		−3.8	LLMGnc_162	−2.1					−2.2
LLMGnc_073	−2.4	−2.1	−2.2	−4.5	−2.4	−4.0	LLMGnc_164		3.0		2.3		−6.2
LLMGnc_075	2.8		−2.1	2.1		−19.1	LLMGnc_165	3.5	2.8	3.4	5.0	2.1	2.7
LLMGnc_076				4.0		−2.4	LLMGnc_175		−2.1	−12.0			−48.6
LLMGnc_079	4.1	−6.2	−280	−2.1		−4.2	LLMGnc_176				6.3		
LLMGnc_080	2.5			2.3			LLMGnc_177	10.4					2.9
LLMGnc_081						−2.4	LLMGnc_178			−2.2			−4.7
LLMGnc_082	7.0					−2.6	LLMGnc_179	2.8					
LLMGnc_083		3.4		2.5		−2.6	LLMGnc_180		−3.1				
LLMGnc_084	2.9			2.9			LLMGnc_182		3.2	−2.1	3.3		−2.0
LLMGnc_085					−11	−4.0	LLMGnc_184			−2.3	−3.2	−2.4	−2.1

*The 110 sRNA genes that are differentially expressed in at least one stress condition, with a cut-off fold change ≥ 2, p ≤ 0.05 and logCPM > 1 are indicated. Blue: upregulated, red: downregulated, color intensity is a measure of fold change. The names of the sRNAs are those that have been previously annotated in L. lactis MG1363, the plasmid-free derivative of L. lactis NCDO712 (van der Meulen et al., 2016)*.

### Most transfer RNAs decrease rapidly after a short pulse of stress

Transfer RNAs (tRNAs) play a crucial role in the translation of mRNAs and it is important for cells to balance their tRNA levels, as well as to ensure optimal utilization of amino acids. Most of the *L. lactis* tRNAs are downregulated under all of the stresses applied. A number of tRNAs, such as NCDO_2402 (Val-CAG) and NCDO_2022 (Lys-TTT), are upregulated under some of the conditions. A distinct tRNA response is observed upon cold stress; seven tRNAs are upregulated by at least 2-fold. Exposure to acid or starvation stress induced the most severe changes in tRNA expression. See Figure 4 for a complete overview of tRNA expression under the various stress conditions.

### Cold stress induces a zinc uptake system

During a 5-min cold stress at 10°C, 412 genes were differentially expressed (Table 1). The most highly upregulated transcripts include those from the *zit* operon, a gene cluster involved in Zn^2+^ uptake and regulation (Llull and Poquet, 2004). Also, expression of the gene *sugE*, encoding a presumed multidrug resistance protein, was increased (~18-fold), as well as genes involved in nucleotide synthesis (*pur* and *pyr* operons) and the *fab* and *acc* operons for saturated fatty acid biosynthesis. Transcripts encoding Cold Shock Proteins A, B, C, and D were upregulated at least 4-fold, as would be expected in cells under cold stress (Wouters et al., 1998). The gene of an uncharacterized protein (Llmg_1848) with high sequence similarity to a bacteriocin in other *L. lactis* species, is located downstream of *cspA* and was also upregulated by a factor of ~10. Downregulation was observed e.g., for the fructose utilization *fru* operon (~18-fold), the lysine-specific permease *lysP* gene (~8-fold) and the ribosomal RNA 5S (~9-fold). Thirty-nine sRNAs from intergenic regions were significantly affected, of which 12 were changed at least 5-fold (see Figure 4 and Table 3).

### Heat stress induces, next to protein chaperone genes, the *arc* operon

**Figure 5:**
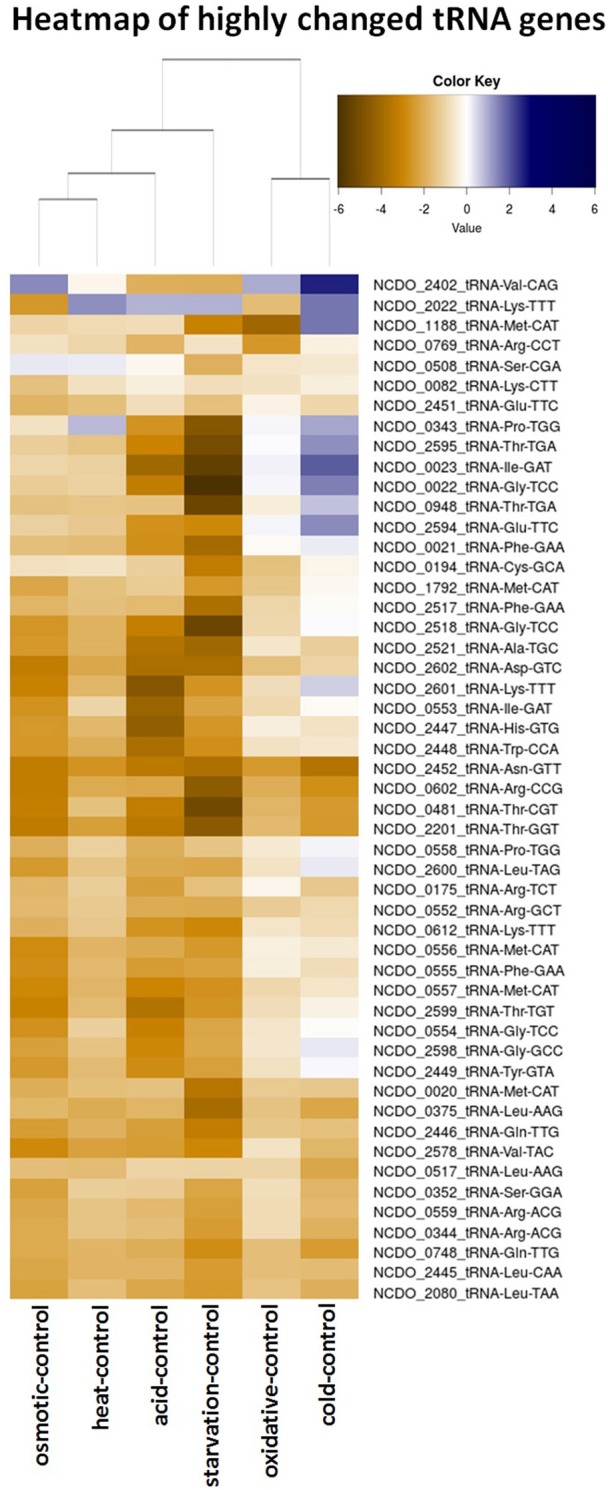
Heatmap of the expression of all tRNA genes from *L. lactis* NCDO712 under the various stress conditions. Expression values are presented in log2-fold, as depicted in the color key.

At high temperatures, proteins may be at risk of denaturation and cells may encounter difficulties in the synthesis of new proteins (Nguyen et al., 1989; Parsell and Lindquist, 1993). The genes of several protein chaperones, such as GroEL, GroES, DnaJ, DnaK, and GrpE are usually upregulated after heat stress. The chaperones aid in protein folding and maturation. Their genes were upregulated ~14–18 times (except *dnaJ*, which was increased ~5-fold) after *L. lactis* was placed for 5 min at 42°C (see Table 2). The Clp protease genes *clpE, clpP*, and *clpB* were also upregulated, as was the gene with an unknown function upstream of *clpE, llmg_0527*. Surprisingly, expression of genes belonging to the arginine deaminase pathway (*arc* operon) were increased ~11-fold, while this operon was downregulated after 30 min of incubation of *L. lactis* IL1403 at 42°C in previous work (Xie et al., 2004). An operon predicted to be involved in the utilization of maltose (*llmg_0485-llmg_0490*), the ribose operon (*rbsABCDK*) and *llmg_1210*, predicted to encode the drug resistance transporter EmrB, were all upregulated. We also observed that the *lacR*-*lacABCDEF* gene cluster, located on the largest plasmid pLP712 of *L. lactis* NCDO712 (Wegmann et al., 2012) and involved in lactose utilization, was upregulated ~3-fold. The heat treatment caused a decrease by a factor of 2–3 of several rRNA species, as well as of most tRNA-transcripts (Figure 5). An operon (*llmg_2513-llmg_2515*) containing a gene for the universal stress protein A (UspA) and two uncharacterized genes was decreased most severely after heat shock, followed in severity of fold change by the multidrug resistance protein B gene (*lmrB, llmg_1104)*. In total, 37 sRNAs were differentially expressed (Table 3). Expression of two of them was decreased over 5-fold: LLMGnc_065 (−6.0) and LLMGnc_079 (−6.2).

### Acid stress induces nucleotide biosynthesis and cysteine/methionine metabolism

Genes for *de novo* synthesis of pyrimidines (*pyr*) and purines (*pur*) were highly upregulated after 5 min of exposure to pH 4.5. Expression of these operons was also seen during starvation and, to a lesser extent, upon cold shock. Among the top upregulated genes were *metCcysK* encoding a cystathionine gamma-lyase and cysteine synthase, respectively, involved in cysteine and methionine metabolism. The genes *llmg_0333-0340* were all affected by the acid stress applied, although they are not transcribed from the same operon. Among these genes are those of a putative methionine ABC transporter system (*llmg_0335-0340* upregulated), and *thiT* (*llmg_0334*) encoding the thiamine transporter (Erkens and Slotboom, 2010) and a putative transcriptional regulator gene (*llmg_0333*) (both downregulated). The *fab* operon, responsible for the biosynthesis of saturated fatty acids (SFAs) for membrane phospholipids, was upregulated. Expression of the operon *llmg_2513-2515* was increased, while these genes were strongly downregulated after heat (see above) and salt stress (see further). The putative Mn^2+/^Fe^2+^ transporter gene *mntH*, which is located on plasmid pNZ712, was upregulated 2.5-fold under pH 4.5 stress. Interestingly, the chromosomal gene *mgtA*, putatively specifying Mg^2+^ transport, was downregulated 3.7-fold. Upregulation was furthermore observed for *llmg_1915-1917* (*ykgEFG*) and for the following genes with predicted functions; *llmg_1066* (unknown), *llmg_1133* (exonuclease), and *llmg_1702* (glutathione reductase). The *fruAKR* operon for fructose transport, conversion of fructose 1-phosphate to fructose 1,6-bisphosphate and their regulation by the FruR repressor (Barriere et al., 2005), was downregulated on average ~28-fold. The *fhuABCD* operon for putative ferric siderophore transport was downregulated by a factor 3–7. Of the 51 sRNA genes of which the expression was significantly changed, only 8 were upregulated; among these was LLMGnc_121, which was exclusively upregulated after acid stress (Table 3).

### Osmotic stress induces chaperones and a putative stress-responsive regulator

Osmotic stress was induced by adding 2.5% NaCl to the cell culture for 5 min. Among the highest upregulated genes are those of an operon (*llmg_2163 - llmg_2164*) specifying a putative stress-responsive transcriptional regulator with a PspC domain (Llmg_2163). Both genes are ~10-fold upregulated; it was also induced upon overproduction in *L. lactis* of the membrane protein BcaP (Pinto et al., 2011) and after exposure to the bacteriocin Lcn972 (Martínez et al., 2007). A deletion mutant of *llmg_2164* was shown to be very sensitive to NaCl (Roces et al., 2009). In *E. coli*, the *psp* operon is induced after application of various types of stresses including salt stress (Brissette et al., 1991). As expected, induction was seen of the genes specifying the glycine betaine ABC transport system BusAA-BusAB (~7- to 8-fold). Some of the responses observed during the exposure to the high concentration of salt were similar to those seen after heat stress. In particular, transcripts encoding the chaperones GroEL, GroES, DnaJ, DnaK, and GrpE were upregulated in the same fold change range. Induction of these proteins has been reported previously for both heat and salt stress (Kilstrup et al., 1997). Both operons for oligopeptide transport were also upregulated under the salt stress applied here.

Genes encoding transporters for various substrates were strongly downregulated, among which the PTS IIA component genes *fruA* and *ptcA*, that of the IIB PTS component, *bglP, lmrB* specifying multidrug resistance protein B (both *llmg_0967* and *llmg_1104*), *msmK*, encoding a multiple sugar ABC transporter and *amtB* involved in ammonium transport. Also, the gene encoding the glycerol uptake facilitator protein GlpF2 was downregulated by ~10-fold, as was the *lac* operon on pLP712. As shown before (Xie et al., 2004), the *potABCD* operon involved in spermidine/putrescine transport and the fatty acid biosynthesis operons *fab* and *acc* were downregulated under osmotic stress. As mentioned above, the *llmg_2513-2515* operon was downregulated. From the 44 significantly affected sRNA genes, the expression of LLMGnc_036 (214-fold down), LLMGnc_118 (75-fold down), and LLMGnc_176 (6.3-fold up) was specifically only changed after salt stress using a threshold of 5-fold change.

### Shaking of a culture of *L. lactis* triggers saturated fatty acid biosynthesis genes

From all stress conditions tested, oxidative stress applied by shaking of the culture resulted in the least number of differentially expressed genes (Table 1). The ones that did change did so with relatively minor fold changes. Among the few upregulated genes were those of the pathway for saturated fatty acid biosynthesis, including *fabT*, the transcriptional repressor of this route (Eckhardt et al., 2013). Downregulation was seen of *arcABD1C1C2, llmg_1915-1917* (*ykgEFG*), and of genes involved in the uptake and/or utilization of maltose, trehalose, and lactose. The heat shock chaperone genes *groEL* and *groES* were downregulated ~2-fold. Unexpectedly, the gene for the manganese superoxide dismutase SodA, an enzyme well-known for its role in oxidative stress, was decreased 2-fold relative to the unstressed control. Twenty sRNAs were affected by at least 2-fold. LLMGnc_019 was downregulated 9.4-fold, while it was upregulated in all the other stress conditions (see Table 3).

### Starvation in PBS resulted in the most dramatic transcriptome changes

Incubation of the cells for 5 min in PBS greatly affected the transcriptome of *L. lactis*, as witnessed by the large number of 150 genes of which the expression had changed significantly, and at least by 5-fold. Different biological functions were switched on or off in response to the sudden absence of basically all nutrients. For example, the operons for the *de novo* biosynthesis of nucleotides, *pyr, pur*, and *car*, were all highly upregulated. Moreover, the genes of several transport systems were upregulated in an apparent attempt to import a (any) carbon source (lactose: *lac* operon, multiple sugar ABC transporter: *msmK*, ribose: *rbs* operon, cellobiose/glucose: *ptcC*), ions (manganese: *mtsA*, zinc: *zit* operon), amino acids (arginine: *arc*, methionine: *metQ*, branched-chain amino acid transporter: *ctrA/bcaP*), and vitamins (riboflavin: *rib* operon). On the other hand, some uptake system genes were downregulated, such as *thiT*, specifying the thiamin transporter, and the *fru* operon for fructose uptake and utilization. Strong downregulation was also observed for the genes of all six cold shock proteins. Dozens of (predicted) transcriptional regulators were affected upon the starvation stress applied here, among which all of the *spxA* genes. Interestingly, the expression profiles were very different. For instance, *spxA* with locus tag *llmg_0640* was decreased ~19-fold, while expression of *llmg_1703* and *llmg_1130* was increased 12.5- and 53-fold, respectively. Notably, the gene for the putative transcriptional repressor CadC, which is located on plasmid pSH73 (Tarazanova et al., 2016), was 4.9-fold upregulated. Besides a strong decrease in the expression of different tRNA genes, also transcripts for ribosomal proteins were downregulated. Starvation changed the expression of 75 sRNAs, of which the majority was downregulated. The nine sRNA genes that were upregulated after starvation were also increased under at least one of the other conditions tested here, with the exception of LLMGnc_060, which was only affected after starvation.

## Discussion

High-throughput RNA sequencing was used in this study to examine the transcriptome changes caused by various industrially relevant stress conditions applied to *L. lactis* NCDO712. Previous studies using DNA microarray- and proteomics technologies have identified genes and proteins involved in the various environmental stress responses in *L. lactis*. However, little to no insight has been obtained so far as to which small regulatory RNAs (sRNAs) and antisense transcripts (asRNAs) are affected by stress, and to what extent. The strand specificity of DNA microarray probes does not allow detection using this technology of antisense transcripts. Also, conventional DNA microarrays usually do not carry tRNA probes and, of course, no probes for as yet undefined transcripts; high-density tilling arrays can detect unknown transcripts. RNA sequencing can be used to uncover all transcripts in an organism at a specific moment in time. It also provides a higher dynamic range for quantitative gene expression analysis than DNA microarrays, provides single-base resolution and suffers less from background noise signals (Wang et al., 2009). In the present study, we have applied a size cut-off of 50 nt to detect sRNAs.

Bacterial cells employed during starter culture or cheese production encounter stress conditions that are similar to the ones applied here, albeit not always as instant and short-lived (5 min induction in our experiments). We chose such a very brief duration of the stressors because we were interested in the ensuing very first transcriptional responses, while in previous studies stress conditions were applied for 10 min to up to 4 h (Sanders et al., 1999). The longer the exposure time, the more secondary effects are activated that obscure the actual first response. The expression of stress-induced genes can quickly build up to a certain level, after which it decreases again. This is, for instance, observed for the *L. lactis hrcA, groESL, dnaJ*, and *dnaK* genes, which all reach a maximum expression level after 15 min of heat shock (Arnau et al., 1996). A study in *Salmonella typhimurium* shows a detailed overview of sRNA expression over time, by sequencing of Hfq-bound transcripts. While some sRNAs were expressed throughout growth, others were only dominant at one specific growth phase (Chao et al., 2012). Growth-phase and stress-dependent expression of sRNAs in *L. lactis* were only conducted for a selection of sRNAs in our previous work (van der Meulen et al., 2016). Here, we focused our analyses on the changes in expression levels of sRNA genes and of tRNA genes. Albeit that the current study was of a fundamental nature, some of the results presented here might ultimately be used in starter culture production or milk fermentation by applying short pulses of stress to the bacteria.

We observed that a number of operons and individual genes were highly affected by three or more stress conditions. For example, the *pur* and *pyr* operons for the *de novo* synthesis of purines and pyrimidines were upregulated after cold, acid and starvation stress. The *fruAKR* was downregulated upon cold, acid, osmotic, and starvation stress. The *fru* operon was previously reported to be upregulated in response to cell envelope stress by the bacteriocin Lcn972 in *L. lactis* (Martínez et al., 2007), and upregulated in *Lactococcus garvieae* after cold stress (Aguado-Urda et al., 2013). Therefore, we could argue that *fru* is highly reactive to different stressors. The *metC*-*cysK* operon was upregulated under all stress conditions applied here except salt addition. The highest effect was observed in acid stress. The *Lactobacillus plantarum metC-cysK* genes were upregulated after exposure to 0.1% porcine bile (Bron et al., 2006), but downregulated by *p*-coumaric acid (Reverón et al., 2012). Genes from the saturated fatty acid biosynthesis pathway (*fab* and *acc*) were upregulated after oxidative, cold and acid stress, while they were downregulated after starvation, heat and salt stress. For cold stress, however, one would have expected to see a decrease in SFAs, to maintain membrane fluidity at lower temperatures (Tsakalidou and Papadimitriou, 2011). The differences observed during oxidative stress might not be caused by higher levels of oxygen, but rather by the shaking that was used to induce oxidative stress. Previously, it was reported that cell envelope stress caused by membrane protein overproduction also affects *fab*. The exact response in this study depended on the identity of the overproduced membrane protein, leading to either an increase or a decrease in *fab* expression (Marreddy et al., 2011). We therefore propose that fatty acid biosynthesis in *L. lactis* is highly adaptive to various stressors and might rapidly fluctuate in time after the stress has been applied.

**Figure 6:**
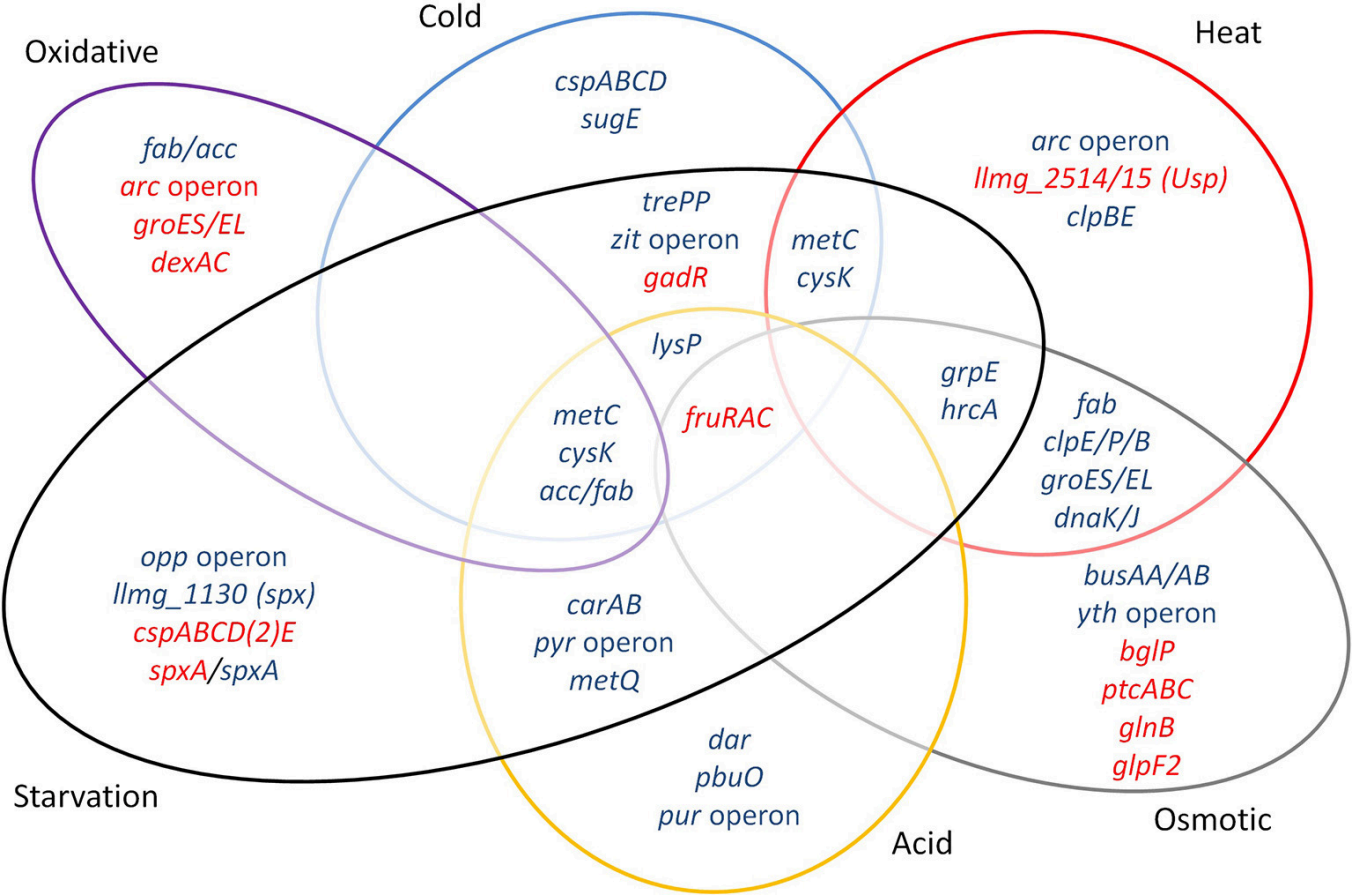
Venn diagram assembled from highly differentially expressed genes and stress-related operons and genes reported previously and observed in this study. Blue font: upregulated genes, red font: downregulated genes.

Major groups of defense mechanisms were significantly induced, despite the short exposure times employed. These include transcripts encoding protein chaperones such as GroEL, GroES, DnaK, DnaJ, and GrpE, which were induced during heat and salt stress. These conditions also induced protease genes such as *clpE, clpP*, and *clpB*. Cold shock induces *cspA, cspB, cspC*, and *cspD* specifying the major cold shock proteins binding to DNA or RNA (Ermolenko and Makhatadze, 2002). During cold stress, the *zit* operon for the uptake of Zn^2+^ was highly upregulated, suggesting that zinc ions play an important role during cold stress, possibly as a cofactor for cold stress-related proteins, and/or have an effect on membrane fluidity. Osmotic stress expectedly induced the expression of the genes of the transport proteins BusAA-BusAB. However, no significant induction of *gadCB* was observed after osmotic and heat stress. Strong downregulation of *glpF2* was observed specifically after osmotic stress, while it was slightly increased after starvation. The glycerol uptake facilitator protein GlpF2 from in *L. plantarum* was shown to facilitate the diffusion of water, dihydroxyacetone and glycerol (Bienert et al., 2013), and could be an important factor for osmotic homeostasis in the cell. The upregulation of the *arc* operon after incubation for 5 min at 42°C was unexpected, since it has been reported before that *arc* decreases upon 30 min of heat stress at 42°C (Xie et al., 2004). This might be explained by differences in the heat exposure times and/or the specific strains used. Also, the arc operon is under complex regulation, with several protein regulators being involved [CcpA, ArgR/AhrC and, possibly, CodY (den Hengst et al., 2005; Zomer et al., 2007; Larsen et al., 2008)], which apparently leads to a rather dynamic expression profile (J.P. Pinto, PhD thesis, Groningen, 2015). Another unexpected result was the decrease of *sodA* expression during oxidative stress, while this gene is usually reported to be strongly induced under these circumstances (Sanders et al., 1995; Miyoshi et al., 2003). The short time of shaking (5 min) did perhaps not allow building up stressful oxygen levels. A more pronounced effect could have been generated with baffled shake flasks or by active addition of oxygen to the flasks, although these conditions would be far from industrial reality. Stress-related operons and genes reported before and observed here are summarized in Figure 6.

The plasmids of *L. lactis* NCDO712 have recently been sequenced and annotated, allowing examining the responses of their genes to the various stresses applied here. Indeed, various plasmid genes were affected under stress, such as the lactose utilization *lac* operon on pLP712, the putative Mn^2+/^Fe^2+^ transporter specified by *mntH* (pNZ712; acid stress) and the gene for a possible transcriptional repressor, *cadC* (pSH73; starvation). Since we applied a very short time of 5 min of stress exposure it is unlikely that these differences were the result of a change in plasmid copy numbers.

Transfer RNAs (tRNAs) are crucial components in translation. The rate of translation of a certain codon is directly coupled to the amount of cognate tRNA in the cell (Varenne et al., 1984), which is a measure of gene expression potential of the bacterial cell. Recently, the tRNAome of *L. lactis* was determined including the positions of 16 post-transcriptional modifications (Puri et al., 2014). Protein-overexpression stress employed in that study led to changes in tRNA expression required for up-regulation of housekeeping genes. An increase in tRNAs that would reflect the codon usage of the gene of the overexpressed protein was not seen. In the study presented here we generally observe a decrease of most of the tRNAs when *L. lactis* is placed under stress. Heat, cold, and oxidative stress seems to affect cellular tRNA transcript levels the least; we noted that cold stress induces the expression of seven tRNA genes. The RNA-seq data did not allow uncovering the actual charging or state of modification of the tRNAs.

Of the 186 sRNA genes currently annotated in *L. lactis* more than half were shown to be differentially expressed in response to one or more of the six different stress conditions that we employed. Since functional characterization has only been performed for a small number of these sRNAs (van der Meulen et al., 2016), only a few conclusions can be drawn as of yet. Some of the downregulated sRNAs might perform housekeeping functions and would not be required under conditions in which cells do no longer grow. On the other hand, sRNAs that are induced might respond to the stressor either to bring the cells into a protective state of slower or no growth, or they might act more specifically in order to overcome the harmful environmental change. Research on the latter group of sRNAs could increase our insights in their functioning during industrially relevant stress conditions and is currently ongoing.

## Author contributions

SM and JK designed the experiments; SM performed the experiments; SM and AJ analyzed the data; SM and JK wrote the manuscript.

### Conflict of interest statement

The authors declare that the research was conducted in the absence of any commercial or financial relationships that could be construed as a potential conflict of interest.

## Supplementary material

Supplementary Information S1 Excel table containing the input files for T-Rex.
